# Being a professional nurse until retirement – a qualitative interview study in Germany

**DOI:** 10.1186/s12912-025-03591-y

**Published:** 2025-07-15

**Authors:** Lisa Schmedding, Theresa A. Forbrig, Johannes Gräske

**Affiliations:** 1 Alice Salomon University of Applied Sciences, Alice Salomon Platz 5, 12628 Berlin, Germany; 2 University Hospital Münster, Albert-Schweitzer-Campus 1, 48149 Münster, Germany

**Keywords:** Nurse’s role, Professional autonomy, Job satisfaction, Personnel retention, Qualitative research

## Abstract

**Background:**

Nurses are crucial to the global healthcare system, yet there is a growing shortage, exacerbated by demographic shifts and the coronavirus disease 2019 (COVID-19) pandemic. The International Council of Nurses projects a deficit of 13 million nurses worldwide in the near future, contributing to missed nursing care and jeopardizing patient safety. Although extensive research has explored reasons why nurses leave the profession, far less is known about the factors that support their long-term commitment and retention until retirement. Hence, study aimed to identify the factors contributing to long-term retention of nurses in the profession, particularly those who stay until retirement.

**Methods:**

This qualitative study used semi-structured expert interviews with 22 nurses in Germany who had at least 30 years of work experience, aiming to explore the personal, organizational, and societal factors that have supported their continued engagement in the profession. A purposive sampling method was used to select participants from various care settings, including acute, long-term, and outpatient care. Data were audio- and video-recorded, transcribed, and analyzed using content-structuring qualitative content analysis. Thematic categories were developed using a structured content analysis approach following Kuckartz, supported by both deductive and inductive coding via the MAXQDA software to identify key themes related to job retention.

**Results:**

The analysis revealed three overarching thematic categories: personal resources and motivations, organizational context and working conditions, and recognition and social value. The first category includes factors such as career choice motives, personal values, and generation-specific influences, all of which are significant contributors to long-term career commitment. The category organizational context and working conditions, including job satisfaction, work-time control, role clarity, and coping strategies, plays a pivotal role in retaining nurses. The third category encompassed the perceived value of nursing work in society, appreciation from patients and peers, and the professional identity fostered by meaningful recognition.

**Discussion:**

This study provides valuable insights into the factors that contribute to nurses’ decisions to stay in the profession until retirement. While many studies emphasize the challenges and negative aspects of nursing that lead to attrition, this research highlights the positive factors that promote job retention. Addressing these factors, such as improving working conditions, ensuring adequate recognition, and supporting personal values and career motivations, could enhance nurse retention strategies. These findings underscore the importance of retention-focused strategies that enhance meaning in work, professional identity, and workplace culture—shifting the policy lens from attrition prevention to long-term engagement.

**Clinical trial number:**

Not applicable.

**Supplementary Information:**

The online version contains supplementary material available at 10.1186/s12912-025-03591-y.

## Background

Nurses are an essential part of the health care system throughout the world. Globally, the World Health Organization [1] states there are 19.3 million professional nurses. Compared with other countries, especially in Europe, the proportion of nurses with a bachelor’s degree is lower in Germany [2]. Even though there is compelling evidence that academic education improves patient safety and outcomes, Germany lags behind nations such as the Netherlands, Sweden, and the United Kingdom, where up to 100% of nurses have academic credentials [3]. In Germany, the nursing profession has traditionally been based on a three-year vocational training program. Since 2003/2004, selected universities have also offered academic nursing degree programs through pilot initiatives. A significant legal change came with the Nursing Professions Act (*Pflegeberufegesetz*) in 2017, which formally established undergraduate academic nursing education as an alternative to vocational training. However, this reform applies only to initial nursing qualification programs. Advanced academic degrees in nursing—such as nursing education, nursing science, and nursing management—have a longer academic tradition in Germany but do not qualify graduates for direct clinical practice. Compared to other countries such as the Netherlands or the United Kingdom, where academic nursing education has been established for decades, Germany introduced undergraduate nursing degrees relatively late. This historical development continues to influence the professional identity and public perception of nurses in Germany today [4]. This lag also stems from a convoluted historical trajectory influenced by gender norms, religious institutions, and disjointed organizational structures, all of which continue to impact nursing’s reputation in society and the workplace [5]. Enhancing the professional identity and visibility of nurses through academic empowerment and structural reform is not only a response to workforce shortages but a prerequisite for sustaining quality care in the future. In recent years, German healthcare organizations have increasingly adopted international frameworks such as the Magnet® and Pathway to Excellence® principles to improve working conditions and nurse retention. These initiatives aim to foster supportive leadership, shared governance, and recognition of nursing competencies, aligning with global efforts to strengthen nursing work environments [6].

The recent coronavirus disease 2019 (COVID-19) pandemic has highlighted the shortage of nurses. In addition, the International Council of Nurses [7] estimates there will be a shortage of 13 million nurses in the near future. A nursing shortage can lead to omitted or delayed care provision [8, 9]. This phenomenon is known as missed nursing care (MNC) [10], which could increase hazards for patient safety, such as mouth care [11], checking equipment [12], and handwashing [13]. Additionally, MNC is associated with the intention to quit the nursing profession [14].

Against this background, many studies have focused on early attrition from the nursing profession. In a Korean study, Lee [15] showed that nurse turnover within the first year was 25%. A study from Sweden showed that 2% of nurses left their profession within the first 5 years after graduating [16]. Besides regular retirement of nurses, a major reason for the nursing shortage is a desire to leave the job. According to a survey of Norwegian nursing homes, 25% of nurses expressed a wish to leave their job, and an additional 25% of nurses were uncertain of whether they wanted to continue in the profession [17]. In a German survey, in all areas of nursing, nearly 40% of nurses indicated they considered leaving their job at least once per month [18]. In a Polish study, about 50% of the nurse respondents from the hospital sector indicated a willingness to leave their profession [19]. In the United States, a survey identified that nearly 30% of nurses intended to leave their job [20]; in an Ethiopian survey, about 61.3% of nurses indicated their intention to leave [21]. Also in the United States, Taylor-Clark, Swiger [22] identified 49% of participating nurses intending to leave the job. However, almost half (44%) of the reason are potentially preventable.

The reasons behind the intention to leave the nursing profession are well known. In a qualitative systematic review using a meta-aggregation, Bahlman-van Ooijen, Malfait [23] identified 11 synthesized categories, including a challenging work environment (e.g., poor working conditions), emotional distress (e.g., work-related stress), disappointment about the nursing reality (e.g., nursing as a second-best career choice), and a culture of hierarchy and discrimination (e.g., feeling subordinate). A large study from the National Sample Survey of Registered Nurses in the United States revealed that burn-out (odds ratio [OR] 2.1) and working 40 hours per week (OR 3.28) compared with working less than 20 hours per week are major reasons to leave the profession [24]. In addition [25], described a lack recognition is associated with the intention to leave the job.

While data on nurse attrition have been well established, far less attention has been paid to the opposite phenomenon: nurses who stay in the profession. In a recent survey from the United States, about 79% of nurses stated that they have considered staying in the profession until their retirement [26]. Data suggest that the reasons why nurses consider staying in the profession until retirement include work-time control, role clarity, and colleague support [27]. Furthermore, job satisfaction among health care workers may predict job retention [28]. However, newer or more comprehensive data are not available, particularly regarding long-term retention. Hence, the present qualitative study shifted the perspective from studying attrition to understanding long-term retention to identify factors that contribute to job retention until retirement.

## Methods

The present study applied a qualitative research design. It was chosen to explore the interplay of ideological and practice-oriented identification with the nursing profession and to examine stabilizing resources that counteract processes of professional destabilization and gratification crises [29]. Participants were selected for interviews to record complex lifeworlds and questions of individual interactions. The use of expert interviews as a qualitative research method is appropriate for this study because process and interpretative knowledge from the action environment, the institutionalized competence of the person, is relevant [30]. Expert interviews are particularly suited for accessing both reflective and practice-oriented knowledge accumulated over long professional careers. In the context of the present study, expert interviews were used to uncover tacit understandings, norms, and personal strategies that have supported retention over decades of work. Furthermore, a qualitative approach allows access to emotional, social, and impact-related dimensions that extend beyond answering predefined questions [31].

### Theoretical framework

This study is grounded in interpretivist constructivism [32], which posits that reality is co-constructed through individual meaning-making and social interactions, shaped by socio-cultural contexts, generational values, and workplace dynamics. According to Tanlaka and Aryal [33], this paradigm emphasizes understanding how nurses actively negotiate their professional identities and career trajectories within evolving societal expectations (e.g., shifting perceptions of nursing and intergenerational differences in work ethics) and institutional practices (e.g., team relationships and leadership styles). This approach prioritizes nurses’ lived experiences as they interpret and navigate their roles over decades, integrating personal values (e.g., altruism and resilience) with external influences such as workplace recognition, media representation of nursing, and policy changes. By adopting expert interviews and content-structuring analysis, this study mirrors the constructivist focus on dialogical processes, where knowledge is co-created through researcher–participant interactions and contextualized within the nurses’ narratives of adaptation and identity formation.

### Setting and participants

Following the principle of “multiperspectivity” in qualitative research, a criteria-based purposive sampling strategy was implemented to ensure topic relevance and adequacy of the sample composition [34]. A total of 21 interviews were conducted, including 20 individual interviews and one double interview with two participants. The double interview was conducted at the request of the participants, who had worked closely together for many years and preferred to be interviewed together for reasons of comfort and security. Both participants met the inclusion criteria and contributed individually to the discussion. Although the interview was conducted jointly, each participant shared their personal experiences and perspectives. Particular attention was paid to interview nurses across a variety of care settings (acute, long-term, and outpatient) and regions of Germany. This approach ensured that the data reflect multiple perspectives on nursing work culture and retention across institutional contexts. To participate in the present study, nurses had have a minimum of 30 years of work experience. The participants were identified based on criteria-based sampling—that is, sampling using predefined criteria to bundle the knowledge of a specific target group [31]. The final sample was selected to reflect both heterogeneity and homogeneity in key characteristics relevant to the research topic. The other inclusion criteria included still working as a registered nurse with at least 3 years of professional training, working with people dependent on receiving care, and provided informed consent. Individuals working in administrative areas were excluded. Personal contacts (followed by the snowball procedure) and advertising on social media (LinkedIn and Facebook groups relevant to German nurses) and in nursing journals (Altenpflege newsletter) were used to recruit participants. The advertisements included a brief description of the study aim, eligibility criteria (minimum 30 years of professional experience, current work as a registered nurse in direct patient care), and contact information for the study team. Interested individuals were invited to contact the research team via email, after which they were provided with detailed study information and a consent form. All responses were screened by the first author to verify eligibility before interviews were scheduled. All nurses who expressed an interest in participating were provided with written information about the aim of the study, data collection, and data protection. Additionally, they were given the interviewer’s name and contact address. No further relationship was established in advance. Although the 26 nurses who received this information provided their written informed consent to participate, four of them withdrew their consent without declaring a reason.

### Data collection

Appointments with the 22 participants were made via email between May and June 2023. The interviews took place throughout June 2023 in different settings (mainly online) and were conducted in German. The interviews were conducted only with the participants; no other people were present. Prior to beginning the interview, the participant provided oral informed consent. Each interview was audio- and video-recorded.

The interviews by LS (a female interviewer) were explicitly guided by the qualitative research interest and adhered to its principles, following the orientation approach suggested by Kruse [35]. A standardized guide was developed for conducting the interview. The use of guideline-based interviews aids in a survey situation through the control and structuring function, with the necessary openness to be able to incorporate the unforeseen [36]. In addition to the opening and closing questions, the research project guidelines contain six thematic blocks, each of which consist of 2–5 main questions that arise from the research questions. The first block of topics deals with the personal professional biography, which offers the interviewees the opportunity to reflect on their original motivation for choosing a career and the situation on the labor market at that time. The second block of topics relates to work organization, which, in addition to the advantages and disadvantages of alternating shift work, also focuses on changes to working time models and the importance of opportunities for further development and advancement. In the third topic block, the past development of work demands in the nursing profession and possible coping strategies are discussed. The fourth thematic block refers to various social aspects that affect professional status and job satisfaction, while the fifth thematic block directly addresses the factors described by the participants as beneficial for their long-term career retention. The nurses are encouraged to go back to their initial professional and training period to draw a comparison with the possible attitudes and ideas of today’s young professionals. The last topic block addresses the assessment of the appreciation and reputation of the nursing profession, which relates to the different role perspectives, material and immaterial factors within the five main questions. The final question that follows should serve as a connection to the initial question and lead to a renewed reflection on what has already been said. It gives the participants the opportunity to share what has not yet been asked and what has been said and to address any outstanding questions or comments on the topic. To ensure all relevant aspects of the expert interviews, all interviews were carried out using the same guidelines [37]. At the beginning of the interviews, a short questionnaire was administered to obtain essential personal and structural background information that is important to the research questions. This information was collected by following the strict data protection regulations [38].

For processing, the interviews were arranged numerically and chronologically. Prior to transcription, the researcher established transcription rules based on previous studies [39–41]. The interview transcriptions were completed between July and September via a writing service hired as part of the research project and made available to the researchers via an online storage facility. Post-interview field notes were also considered. All transcripts were stored securely and made available to the researcher via a protected online platform. Due to time restrictions, explicit validation of the transcripts by the participants was not possible.

### Data analysis

**Fig. 1:**
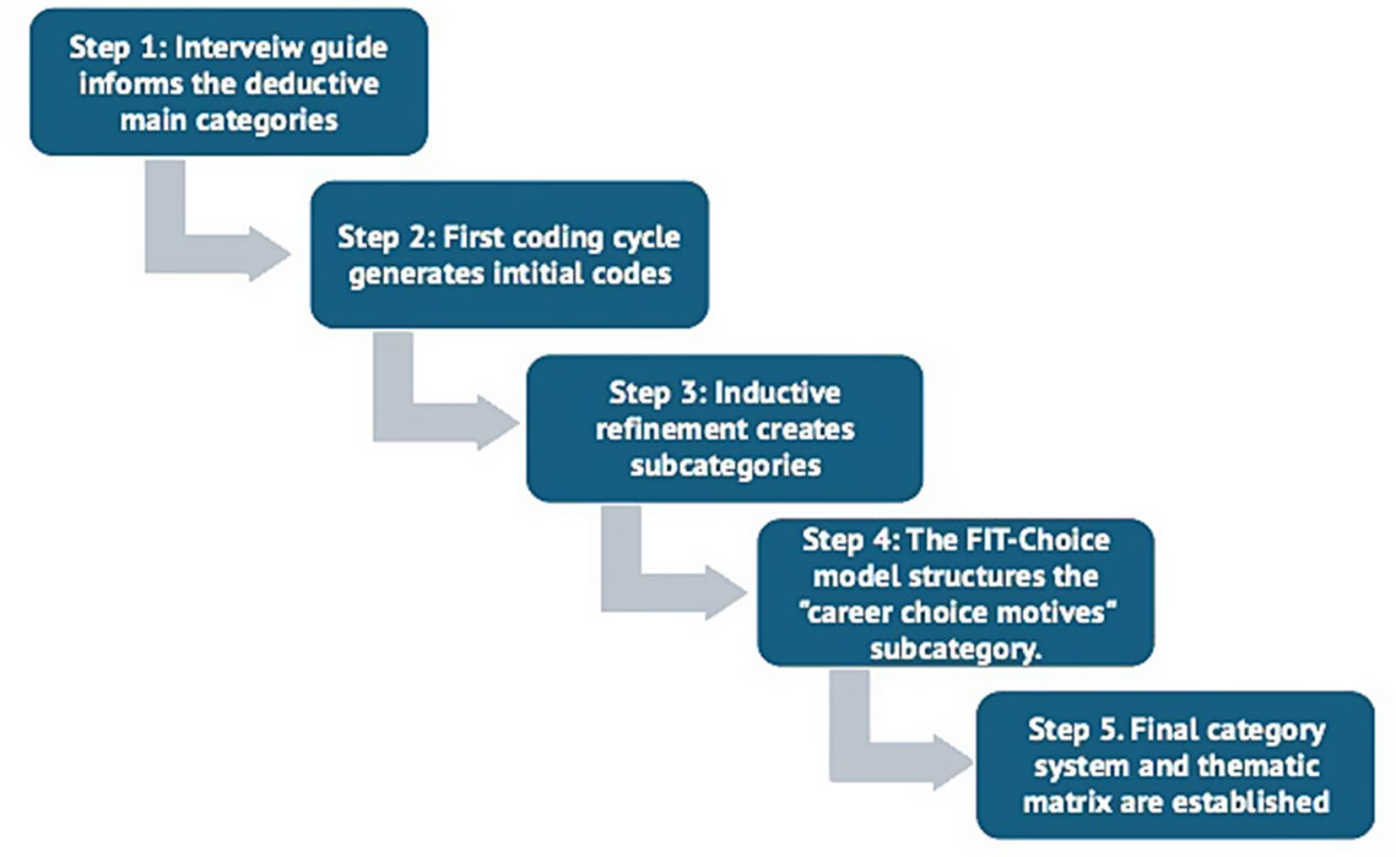
Category development process

Data analysis followed the seven-phase model of content-structuring qualitative content analysis according to Kuckartz [42], which includes initial text work, category formation, coding, and interpretation. Coding was done by LS, a well-experienced registered nurse (RN) and research assistant. During the analysis steps (see Fig. 1: Category development process), she was engaged in self-reflection to identify how her perspective could bias data analysis. After the interviews were transcribed, the text material was evaluated using content-structuring qualitative content analysis according to Kuckartz and Rädiker [43]. Qualitative content analysis offers the possibility of identifying and conceptualizing content aspects based on the data material and consequently describing them systematically using a categorization system (Schreiner [44]. The researcher is guided by the flow chart of the partially computer-aided, seven-phase content analysis.

The MAXQDA software was used for systematic, structured, and transparent analysis for the initiating text work during the first phase and for data evaluation [43]. In the subsequent phase of the Kuckartz flowchart, an initial coding process took place in which the entire text was assessed sequentially and assigned to categories [43]. Initially, seven main thematic categories were formed deductively based on the structure of the interview guide and aligned with the study’s research questions. The deductive formation of three main thematic categories was based on the main factors identified in the background for why nurses leave the nursing profession. Due to the thematic diversity, the first topic block of personal characteristics was divided inductively into three categories: career choice motives, values, and generation-specific influencing factors. The subcategories for the theme career choice motives were developed with reference to the factors influencing teaching choice (FIT-Choice) model by Watt and and Richardson [45], including social influences, biography, social and financial benefits, self-perception, personal gains, and intrinsic motivation. The initial category ‘career choice motives’ was deductively derived from the interview guide, as career choice was explicitly addressed in the interviews. However, the subcategories within this theme were developed inductively during data analysis based on the diversity of participants’ narratives. These subcategories reflected nuanced aspects of career choice motivation, such as social influences, biographical factors, intrinsic motivation, and personal gains. Although the FIT-Choice model was originally developed for the teaching profession [45], it has also been applied in nursing education contexts [46]. Therefore, it provided a useful conceptual framework to structure the subcategories, while still reflecting the lived experiences of the participants. This approach reflects the combined deductive–inductive strategy of qualitative content analysis: while the main thematic areas followed the structure of the interview guide, the deeper understanding and differentiation of participant responses emerged through iterative reading and coding of the interview material. The second topic block on working conditions reflects aspects of job satisfaction as well as coping and prevention strategies. The final main category is appreciation and recognition with the subcategories social perspective, perspective of the nursing professional group and media reporting. All identically coded text passages were assigned to the corresponding categories, after which the subcategories were assigned to them inductively in the following phase (i.e., derived from the material to be examined). When forming subcategories, the principles of economy and manageability should be followed to proceed in as differentiated a way as possible. The content-structuring change between deductive and inductive coding as part of qualitative content analysis has been described as characteristic and advantageous for the coding process [44]. To illustrate and define the subcategories formed, example quotations, also known as anchor quotes, from the interview transcriptions were listed in the coding guide [43]. The quotations are presented with anonymized interview numbers (e.g., I6, pos. 8). All quotes were translated from German by the research team and edited for clarity where necessary, without altering the meaning. In total, 1,140 coded segments were identified, forming a comprehensive category system which was then transferred into a thematic matrix for structured analysis. The second coding refers to the entire material and assigns the differentiated categories to the text passages that were previously coded with the main category. The final phase of the content structuring content analysis involves complex analysis, evaluation, and interpretation of the categories. According to Kuckartz and Rädiker [43], various forms are available for evaluating the topics that have been coded. The most common form of category-based analysis, which was used in the present work, behaves interpretatively along the main categories. This final phase involved categorization as well as the interpretation of deeper interrelations within and between categories, reflecting the complexity and subjectivity of the respondents’ experiences.

### Researcher reflexity

The first author’s professional background in nursing shaped the research process in both advantageous and challenging ways. As an insider to the field, the first author possessed contextual knowledge and practical understanding that facilitated access to participants, the formulation of relevant questions, and the interpretation of nuanced professional experiences. At the same time, this proximity to the field required continuous self-reflection to maintain analytical distance and prevent assumptions from influencing data interpretation. In line with established discussions on insider research, this dual perspective proved particularly valuable in addressing the underexplored question of what supports nurses to remain in their profession, ensuring practical relevance while striving for methodological rigor.

## Results

The sample comprised 22 nurses, 18 of whom were interviewed individually, and 2 of whom were interviewed together. No repetition was needed. The mean duration of the interviews was 25:24 (range: 12:28–43:11) minutes. Table 1 presents the main characteristics of participants according to the sociodemographic questionnaire. Data saturation was reached after approximately 18 interviews, with no substantial new themes emerging in the final four interviews. This was confirmed during coding and discussed among the research team.

**Table 1:** Characteristics of the participants

	*n* = 22
Age in years, mean (SD)	55.4 (5.7)
Sex, n (%)	
Female	20 (90.9)
Male	2 (9.1)
Nursing field, n (%)	
Hospital care	16 (72.7)
Outpatient care	4 (18.2)
Long-term care	2 (9.1)
Work experience in years, mean (SD)	36.6 (6.0)
Years in current job, mean (SD)	24.3 (11.3)

SD, standard deviation

Data analysis revealed three main categories: personal characteristics, working conditions, and appreciation and recognition. Multiple subcategories were identified within the main categories (Table 2). The following results are presented by the main categories and illustrated using representative quotes. Note that the categories are not mutually exclusive: The individual narratives often intersected across domains, reflecting the complexity of retention experiences.

**Table 2:** The main categories and their subcategories

Main categories	Subcategories
Personal resources and motivations	
	• Career choice motives
	• Values
	• Generation-specific influencing factors
	• Resources
Organizational context and working conditions	
	• Job satisfaction
	• Coping and prevention strategies
Recognition and social value	
	• Social perspective
	• Perspective of the nursing professional group
	• Media reporting

### Personal resources and motivations

According to the participants, the nursing profession is complex and multi-layered, and long-term retention in the profession depends on various influencing factors and resources. The participants described, influenced by generation-specific characteristics and values, areas of tension that have arisen over the course of their careers between their professional self-image as well as their former motivation to choose their career and the experienced supply realities of increasing work demands. In addition to the discouraging and encouraging influences generated by the social environment, biographical experiences, financial and social aspects, as well as primarily intrinsic values and self-perception have a motivating effect on the choice of career. The discouraging and the encouraging attitude as well as increasingly occurring family dispositions strengthen the motivation to choose a career behind the intrinsically based self-perception and conviction, which, in summary, represents a sustainable resource for long-term career retention:

My parents said that when I was a child... I always made a bonnet like that, because the deaconesses used to wear bonnets like that, so I then made the bonnets for myself and some kind of white capes, (.) and was I then practically, yes, ....the angel and then I vaccinated and looked after all the dolls and toys, so (.) that was somehow in me and I couldn’t imagine anything else than going into care. (I6, pos. 8)^1^

The statements “in my head [was] actually just nursing” (I4, pos. 10) and “that was somehow in me” (I6, item 8) also reflects the old professional ethic of being born for nursing the lasting euphoria that goes with it becomes clear: “I have ALWAYS wanted to be a nurse... I NEVER asked myself what I actually wanted to do for a living, that was ALWAYS rock solid for me, (.) I want to be there for others, I want to help, ... this motivation is actually still there, so that’s really my calling, I love my job” (I19, pos. 12). This narrative reflects an old professional ethos and carries potential risks—such as self-exploitation, blurred boundaries, or problematic role expectations. The perceived “HAPPINESS” (I3, pos. 44) of training and the workplace due to the strong competitive situation of the baby boomers has had a formative effect on the nursing self-image, which is partly responsible for staying in the profession for many years:

If you can choose things, then you always pick the best ones, (.) and you part with the ones that are too weak, yes. That’s how it was (...) we now expect performance. [...] if you look at/(.) what the company has planned, right? The expectations of the company were not met, (.) then there were warnings, there was not much questioning.

(I3, pos. 42)

Furthermore characteristics such as determination and hard work are very pronounced in the participants. The desire for independence and change has influenced their choice of profession and determines the characteristic of assertiveness and perseverance, which the participants perceive as a resource. The labor market situation and the resulting pressure to perform creates an increased drive and professional commitment to remain in the nursing profession.

### Organizational context and working conditions

Job satisfaction, which comprises various aspects, is one of the most central aspects for staying in the nursing profession for a long time. In essence, the participants measure the attractiveness of an employer primarily based on the available support and further development opportunities, in particular, individual support within the company contributes to long-term loyalty:

... When you’re young, you say, “Oh, I’ll stay for two years and then I’ll move on again and move on again.” (.) Yes, and I was just, um, lucky or interested, um, (.) I was met with the fact that they said “Well, we can = we can promote them too”, right? But supporting also means you have to decide whether I want to change quickly or do I really want this support. I decided to support it and had/have experienced a lot of good things from it.

(I3, pos. 10)

In addition to the support offers, the attractiveness of workers also depends heavily on the leadership style as well as “the climate” (I2, pos. 20) in the company and the behavior of the managers: “But I [have] been very lucky and that I think that was also the decisive point why I have been in nursing for so long now, (.) being able to experience a very, very warm hospital (...)” (I16, pos. 10). The ability of managers to learn depends on their leadership skills and is a central influencing factor for remaining in the profession, which can influence working conditions through role model function, “transparency” (I17, pos. 72), and open, solution-oriented communication. Furthermore, the participants unanimously assigned the role of the team a particularly high level of importance in the nursing profession: “Well, I think the TEAM is the most important thing” (I7, pos. 26). A statement from one of the participants—“If I have a team where I don’t feel comfortable, then um (.) I have to leave there. (.)” (I1, pos. 54)—illustrates the high relevance of the team in the nursing profession, which strongly influences job satisfaction and motivation as well as the decision of whether to stay or leave the company or profession. The family comparison of the team mentioned several times illustrates the central role in the eyes of the participants, which goes beyond pure professional cooperation and represents a resource for both work and private life that is “indescribable [and] priceless” (I2, pos. 118) is:

[The] be-all and end-all, my second family. (.) The team is simply a second family, (.) it has ALWAYS been like this, over the years, no matter where I have worked, (.) the team has always (.)/yes, that was the driving force. ... togetherness (...) playing and supporting each other, ... always of great importance.

(I6, pos. 30)

According to the participants, people-oriented nursing activity itself is another beneficial factor for job satisfaction and thus for staying in the nursing profession for a long time. The loss of patient contact through exercising a management position—shown in the comparison refers to the positioning being on “the best side (.) of the bed” (I5, pos. 66)—reflects the strong connection to the professional understanding. In addition to the relationship work, the feeling of being needed and “having done something good and helped people” (I4, pos. 38), positive reflection, varied work, and professional competence are seen as central, motivating factors that influence job satisfaction:

And (.) yes, because I just/I = I like the job, I just have a lot of fun with it, so this (.)/such trifles/making people happy with trifles, on the other hand also this adrenaline rush when you work in an ICU and someone you thought ‘Oh shit, he won’t make it’ (.) then ends up saving someone’s life or someone who is bleeding horribly after an operation and you saw it quickly enough or something like that, (.) and you were able to save him, that’s just great, (.) that’s fun.

(I7, pos. 58)

The participants described “the feeling of doing something meaningful” (I4, pos. 38) as lasting consistently over at least 30 years of employment. They notes that fulfilling professional meaning through substantial work for patients is conducive to remaining in the nursing profession. As a result, 12 of the 22 respondents would choose the nursing profession again “without any ifs or buts” (I6, pos. 56): “Yes. ALWAYS. (.) ALWAYS. There is no other job for me than nursing” (I1, pos. 30–32). When asked why there was no alternative, the participant replied as follows: “(sighs) Because nursing is a profession, (.) they always say that nursing is a calling” (I1, pos. 30–32). For nurses, working in the nursing profession means a purpose that goes beyond financial income and results from a high level of personal identification with the professional tasks. This personal identification in turn helps the participants to stay in their job for the long term, which creates benefits for both their private and working lives. According to all participants, alternating shift work is characterized by high flexibility, variety, and adaptability of private and working life and overall has more advantages than disadvantages. Therefore, the coping strategies that require a long stay in the nursing profession are generated by both the given independence and autonomy in the nursing profession. However, some of participants also reflected on their highly felt personal identification with the nursing profession: “[The nursing profession had] always been very important, sometimes in retrospect I think perhaps it was too important” (I16, pos. 40). This statement shows the difficult separation of professional and private life resulting from the high level of professional identification, which in turn justifies the use and development of various coping and prevention strategies by nurses. These strategies are beneficial for remaining in the nursing profession for the long term and are central to maintaining 1’s health. Thus, professional emotional demarcation is a coping strategy that promotes long-term professional retention. The participants realize this based on their ability to learn from experience and the ability to view their own reflection, mindfulness and awareness of their own health as well as boundaries in both private and professional life as a resource: “You have to tell people that they do a good job, absolutely. (.) This is important” (I3, pos. 48).

### Recognition and social value

Based on the interviews, the general reputation of the nursing profession depends heavily on the reputation of professional nurses. Indeed, one participant stated: “Nursing itself has also ruined its own reputation” (I2, pos. 134). Thinking appreciatively about one’s own work also influences 1’s external reputation: “We as nurses know how important our job is, if we have lost this knowledge, then we have to question ourselves” (I1, pos. 60). A nurse’s reputation and external image play a central role in ensuring that they remain in the nursing profession in the long term. According to the participants, media reporting offers both opportunities and challenges for the development of the nursing profession. The multiple references to the COVID-19 pandemic point to the resulting opportunities: “There was the time before Corona and there was the time after Corona, (.) and that is already on/so the profession is now visible differently” (I5, pos. 84). According to the participants, this means there is a chance of using empty phrases such as “Oh, you work in nursing, ah, (.) I couldn’t do that, right?” (I3, pos. 50) as well as the rationalization to prevent nursing activities from being based on the stereotypes of “going to the toilet, passing food” (I3, pos. 50) and instead to impart knowledge about “what constitutes care and [what] care is and how COMPLEX this area of responsibility is” (I3, pos. 50). The public negative portrayal of nursing through media reporting is partly responsible for this, as stated by a participant from the long-term inpatient area:

Of course, there are (...) always black sheep and there will simply be black sheep and also when Mr. Wallraff [a German investigative journalist] always talks about it and um (.)/then = then my hair stands on end and I ask myself whether = if that’s even the case is still possible because we simply live different care in everyday life.

(I6, pos. 46)

The above statement refers to the media exposure of the abuses in nursing homes, which, according to the participant, creates a negative, blanket image of the nursing profession and defames the actual efforts and achievements. It is clear from the interviews that media reporting affects society’s perspective and is therefore a central influencing factor for remaining in the nursing profession. According to the participants, the role of “appreciation is generally perceived as incredibly important” (I7, pos. 52) in the nursing profession. One participant described this phenomenon in relation to “respect (.) for the work, (.) for [the] tasks and also (.) for the patient [as] very important” (I5, pos. 64). However, the role of 1’s own esteem as a nursing professional is one of the most central influencing factors for long-term retention: “In general, one often wishes that the esteem was greater, (.) But what is more important is that I see myself in “I feel good about my job (.) and therefore ... this self-satisfaction is much more important than satisfaction from outside” (I4, pos. 86). With this statement, the participant alluded to what she sees as a lack of appreciation from society. The participants viewed social and political appreciation as a less relevant reward or influencing factor for staying in the nursing profession compared with their own appreciation. In the eyes of the nursing professionals, the “RIGHT appreciation” does not correspond to “applause or (...) the 1,500 Euro Corona bonus” (I6, pos. 46), but rather to recognition by the company, “from the managers” (I6, pos. 46) and “from the immediate environment” (I2, pos. 126). In contrast, the participants described positive feedback from patients as well as patient satisfaction as central reward factors for professional activity, which have a beneficial effect on long-term professional retention: “I have enjoyed doing it (.) all my life, (.) and (.) I think there is hardly any other profession in which you can get so much positive feedback, (.) and I have always received plenty of it” (I4, pos. 36). The appreciation of patients is a central influencing factor for long-term employment:

Show me a job where I can make people happy by (.) shaking up the blankets, okay? (.) Well, that’s how it is (.), you know, when you go to the patient and say, “Oh man, your blanket is so crumbly, should I shake it up for you,” and people are happy, (.) There’s no job like this anywhere else, is there? So I don’t know of any now.

(I7, pos. 56)

**Fig. 2:**
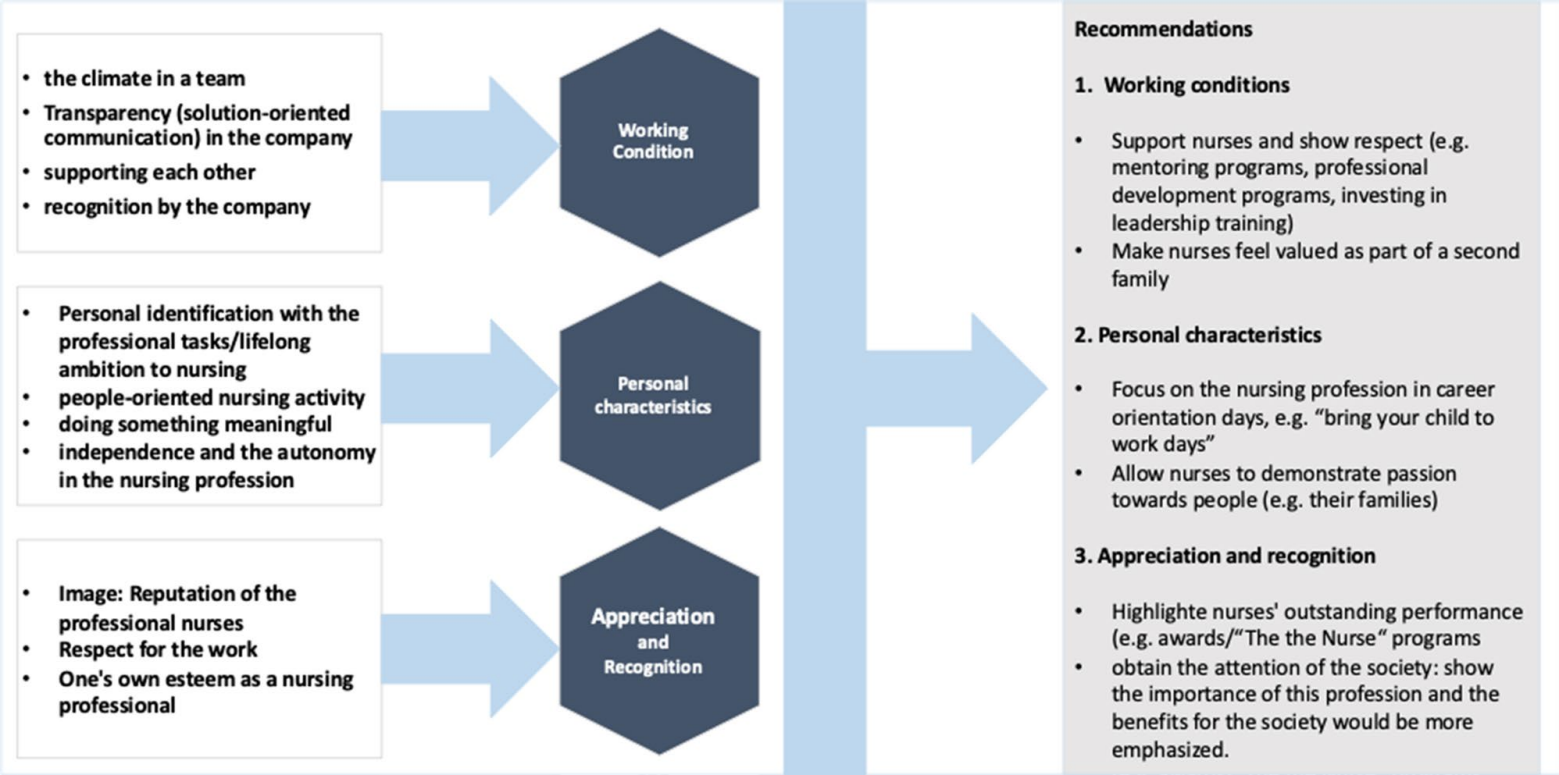
Results and recommendations

In summary, the interviews revealed that in terms of appreciation, intangible and interpersonal influence and reward factors in particular keep nurses in their profession. The influencing factors are based on mutual respect, trust, interest, and attention and apply to all professional and private relationship constellations in the nursing profession. The care and health facilities as well as leadership skills in particular influence long-term retention. According to the participants, the media and public representation of the nursing image also has a significant influence on whether they stay in or start a career. The most important results and the recommendations derived from them, combined with the aim of strengthening the professional identity and visibility of nurses, are summarized in Fig. 2: Results and recommendations.

## Discussion

Given the global shortage of nurses, understanding what keeps experienced nurses in the profession is critical. This study identified three overarching factors that contribute to long-term retention: personal resources and motivations, organizational context and working conditions, and recognition and social value.

### Personal resources and motivations

For nurses staying (almost) until retirement in the nursing profession, becoming a nurse was the only option for their professional career. Many of the participants described a lifelong ambition for nursing, often starting with early childhood experiences. Participants frequently described nursing as a calling or lifelong mission, reflecting a strong personal identification with their work. While this narrative resonates with the historical professional ethos of care and meaning, it also carries risks—such as self-exploitation, blurred work-life boundaries, and unrealistic role expectations. As Yam [47] and Pfabigan and Rappold [48] note, critical and feminist nursing scholarship has long debated how such idealized self-images can obscure the need for fair working conditions, professional recognition, and boundaries between personal identity and occupational role. Our findings reflect this tension: the values that motivate nurses to remain in the profession coexist with systemic challenges that may exploit these very values. A more nuanced understanding of nursing as a complex, evidence-based profession—as opposed to a purely altruistic calling—is essential for supporting sustainable career paths.

These experiences were preserved by values and influences of both family and society. This insight could be used to attract the attention of young children to the nursing profession and lead to long-term retention. Children could be attached to the nursing profession to promote an early interest and respect for nursing. Relevant stakeholders should consider creative opportunities. This approach could include programs where children observe or engage with the healthcare environment, such as hospitals or nursing homes. It is important that nothing could harm the children: Their interaction should be educational and in a safe environment. Interactive days for schools and kindergartens may positively influence children’s perceptions of nursing by making the diversity and career possibilities of the profession visible. However, such initiatives should be critically reflected upon, as they risk reinforcing traditional gender stereotypes that frame caregiving as a predominantly female domain. Broader societal and educational efforts are needed to present nursing as a career open to all genders and to promote its complexity and high level of responsibility. This is not only a task for individual organizations but also a societal and political responsibility, requiring public campaigns, educational reforms, and role models that challenge stereotypical images of the profession. Even interactions with medical equipment in a safe space is considerable. These experiences could create the desire in children to start a nursing career. Moreover, such initiatives could introduce narrative workshops in which nurses narrate their personal stories and the gratification they draw from their job. This can foster a sense of pride and prospect among children. An alternative approach would be to introduce variations of Teddy Bear Hospitals [49] but centering on nursing. This idea is aimed at familiarizing young children with the medical profession, which could in turn evoke aspiration in children to become a nurse.

Moreover, such institutions can even hold educational workshops where children can acquire fundamental caregiving skills—appreciating the significance of hygiene. To add a bit of excitement, they could participate in make-believe games of nursing duties suitable for their age group. These events would aim to be fun and knowledge based, hopefully unforgettable, leaving behind imprints that may steer interest toward nursing from an early age.

Introducing these opportunities in the workplace has dual benefits: child development and fostering stronger relationships between nurses and their work environment. This would allow nurses to demonstrate their passion. Moreover, it would serve as an engaging avenue for work-life balance where nurses can share enjoyable and quality time with their children. This should not only be supportive but also educative in nature.

A child-friendly atmosphere can be cultivated by hospitals and nursing homes with such programs. This could help children view nursing in a positive light and potentially as a rewarding career choice. The novel approach is aimed at addressing the rising need for healthcare staff not only through creating innovative programs but also by inspiring young minds through role models from nursing. These interviews underscore the role of professional identity formation early in life, consistent with the literature on motivation and career commitment [45]. Intrinsic motivation, self-efficacy, and value congruence emerge as central retention drivers. Nevertheless, it is not just a task at company level, it is a task for society as a whole and a political task to strengthen this diversity, this high level of responsibility of the nursing profession and thus to strengthen the nursing profession as a whole.

### Organizational context and working conditions

Previous investigations have found that working conditions are one of the major reasons to leave the nursing profession [23]. The present study identified key elements crucial for nurse retention: support and growth prospects.

Every company offers individual support for nurses; however, these offers are implemented weakly. Mentoring programs for new graduates represent a promising approach to improve job satisfaction and retention in the job [50]. These programs allow experienced nurses to guide newcomers and to provide essential career support and professional wisdom. They support professional growth and can foster strong interpersonal bonds that mirror the team-based culture identified in the present study. Such programs are also considered to be beneficial to mentors [51] and therefore are one way to develop and use the skills of experienced nurses. Thus, healthcare organizations should implement comprehensive professional development programs, including continuous education, certification courses, and advanced training workshops for all experience levels.

A positive work culture, shaped by the appropriate leadership approach and team dynamics, significantly influences job satisfaction and retention. It is critical to invest in leadership training programs for managers and supervisors, focusing on transparent communication and supportive leadership skills. These findings correspond with recent transformation efforts in German hospitals adopting Magnet® and Pathway® principles. Kleine, Köppen [6] identified similar retention-relevant factors such as supportive leadership, professional autonomy, and recognition. These culture change processes reflect broader organizational strategies aimed at improving nurses’ working conditions and strengthening retention in the German healthcare system. There is evidence that different leaderships correlate differently with job satisfaction. Specchia, Cozzolino [52] reported that the transformational style shows the strongest positive correlations; the authentic, resonant, and servant leadership styles also show good results. Genuine cooperation among colleagues, rather than typical team-building events, creates a positive work environment. This includes professional appreciation and close interpersonal connections, making nurses feel valued as part of a second family. Supporting group cohesion with team-building is promising endeavor [53]. The Magnet® approach seems to be more comprehensive because it intends to create a new culture, which is superior to non-magnet hospitals [54]. However, there is a lack of evidence regarding whether this approach is suitable in nursing homes.

### Recognition and social value

Recognition programs demonstrating appreciation for staff contributions are essential: They boost morale and reinforce organizational commitment. These can take various forms, such as awards or public acknowledgments, highlighting nurses’ outstanding performance in different capacities. However, recognition efforts are most effective when personalized and integrated into daily practice rather than delivered through one-off symbolic acts. Burke, Jablonski [55] described the program at the University of Pennsylvania Health System and reported a longitudinal positive impact through recognition. Another well-established form of recognition is the DAISY Award of meaningful recognition, with about 4,600 participating health care organization worldwide [56]. Furthermore, “Thank the Nurse” programs, for example, flowers given on birthdays or any anniversaries, are essential for recognition. However, recognition and respect in society has been investigated much less frequently. A commonly known example is “Clap for Heroes” during the COVID-19 pandemic, although this could not be established to show respect to the nursing profession. Additionally, the image of nurses, as presented by the media, is predominantly negative [57]. The first step to obtain the attention of society is to get more a more realistic image of nurses. Increasing the public’s awareness of what nurses do would improve working conditions because the importance of this profession and its benefits for the society would be highlighted. Beyond institutional efforts, the participants emphasized that meaningful interpersonal appreciation—from patients, peers, and direct supervisors—was more impactful than public campaigns or bonuses. This insight is consistent with findings from Sweeney [56] on the importance of sustained, context-sensitive recognition programs.

Beyond recommendations for individual nurses and healthcare organizations, the findings also highlight the need for broader public and educational policy reforms. Participants’ experiences point to systemic challenges in the recognition of nursing competencies, professional development opportunities, and sustainable career pathways. Therefore, policy measures—such as the planned Nursing Competence Act (*Pflegekompetenzgesetz*)—are urgently needed to strengthen nurses’ professional autonomy and role clarity. Additionally, reforms in nursing education policy should further expand academic qualification pathways and ensure that leadership, research, and advanced practice roles are sustainably embedded in career frameworks.

The results should be interpreted in the context of Germany’s structural lag in nursing academic and professional development, even though the study concentrated on organizational and individual factors for nurse retention. The limited scope of professional autonomy and the sluggish pace of educational reform in Germany may limit long-term career satisfaction and identity development in contrast to nations such as the United Kingdom or Sweden, where academic qualification is the norm. The participants’ strong reliance on informal recognition and intrinsic motivation over formal or systemic professional rewards may also be explained by this context. In addition to strengthening a person’s professional self-concept, closing this gap through more expansive academic pathways and more clearly defined advanced roles may also help retain employees in the long term by increasing their career flexibility, prestige, and recognition. As this study demonstrates, identity, appreciation, and purpose are tightly interwoven—strengthening all three requires not organizational as well as systemic reform.

In sum, long-term retention in nursing is the result of a dynamic interplay between internal commitment, supportive workplace environments, and a sense of professional value. These findings call for targeted strategies that integrate career development, leadership support, and everyday appreciation as core components of retention policy. Future research should explore how these factors interact in different care settings and across generations to inform sustainable retention strategies.

## Limitations

This study offers important insights into the factors supporting long-term nurse retention; however, several limitations must be acknowledged. First, while the participants were recruited from hospital, long-term, and outpatient care settings, the sample was weighted toward hospital-based nurses. This imbalance may limit the transferability of the findings to other care sectors such as home or geriatric nursing. Second, the use of snowball sampling may have led to the inclusion of participants with similar professional values or social networks, potentially narrowing the diversity of perspectives. Although efforts were made to ensure variation in age, region, and care setting, the sample cannot be considered representative of the German nursing workforce as a whole. Third, the study was conducted in Germany, and the findings may be context-specific due to cultural, structural, and policy-related differences in healthcare systems. Further cross-national research is needed to explore whether similar retention factors apply across healthcare environments. Fourth, the transcripts by the participants were not validated. While this approach is considered to improve data in qualitative research, it was not feasible in the present study due to time restrictions. The lack of this validation could have influenced the results and their interpretation. Finally, as with all qualitative studies, the subjective interpretation of data by the researchers may have influenced the analysis. Strategies such as reflexive journaling and intercoder agreement were applied to enhance trustworthiness, but complete objectivity cannot be assumed.

## Electronic supplementary material

Below is the link to the electronic supplementary material.

Supplementary Material 1

## Data Availability

The datasets used and analyzed during the current study are available from the corresponding author upon reasonable request.

## Abbreviations

COVID-19: Coronavirus Disease 2019
ICN: International Council of Nurses
MNC: Missed Nursing Care
RN: Registered Nurse
WHO: World Health Organization

## Footnotes

1 All verbatim quotations were translated from German into English.
