# Supplementary material for: Being a professional nurse until retirement – a qualitative interview study in Germany
Source: BMC Nurs. 2025 Jul 15;24:926. doi: 10.1186/s12912-025-03591-y (PMC12265344; doi:10.1186/s12912-025-03591-y)
Supplement: Supplementary file 1 — Supplementary Material 1 [file 12912_2025_3591_MOESM1_ESM.docx]

**Interview guide**

**Introduction**

To begin with, it would be very helpful for me if you could give me a brief outline of your area of work.

Optional follow-up questions:

- Please also tell me something about your day-to-day work
- Which area of nursing are you working in? (acute, long-term or outpatient care)

**Topic block 1: Professional biography**

1 If you think back to the very beginning of your professional career, what motivated you to enter the profession?

2 If you continue to think back to the beginning of your professional career, what was the general job situation in nursing?

- How many years have you been in the profession?
- How long have you been working in your current field/with the same employer?

Optional follow-up questions:

- What impact did the choice of job have on you and your family?
- How did you deal with these effects at the time?
- How did you experience other people involved or acquaintances in this situation?

**Topic block 2: Work organization**

1 Looking back on your entire career, to what extent do you see advantages and disadvantages in (alternating) shift work?

Optional question:

- What factors have influenced the changes in working hours?

**Topic block 3: Development of work requirements in the nursing profession**

1 When you think about your everyday working life, what changes have you noticed in recent years?

2 How do you cope with challenges for yourself?

**Topic block 4: Social aspects**

1 What aspects can you think of that make you go home satisfied at the end of the working day?

2 What role does your job play in your life?

**Topic block 5: Development of employment in the nursing profession**

1 You yourself have been working in the nursing profession for more than 30 years. When you look back on your years in the profession, what has contributed to you staying in the nursing profession until now?

Optional follow-up questions:

- What is it like for you to work in the care profession as a caregiver in your age group?
- What role does your own attitude to work play for you?
- How important are contractual conditions/securities?
- How important is the working environment?

**Topic block 6: Appreciation and reputation**

1 When you think back to your early days as an examined (pediatric) nurse, how did you feel about your own job satisfaction compared to today?

Optional follow-up questions:

- How would you describe the relationship between pay and working hours?
- What role does your team or the team feeling play in this?

2 What role does appreciation for your profession play for you in general?

- From your personal environment
- From society/politics
- In the form of remuneration

3 To what extent has the perception or reputation of the nursing profession changed over the last few decades?

4 Finally, which aspects do you consider most important for staying in the nursing profession for many years?

**Final question**: Would you choose the profession again today?

**Conclusion:**

We have reached the end of the interview. Is there anything else you would like to say or ask?

Thank you very much for your participation!
